# Sweet Taste as a Predictor of Dietary Intake: A Systematic Review

**DOI:** 10.3390/nu11010094

**Published:** 2019-01-05

**Authors:** Sze-Yen Tan, Robin M. Tucker

**Affiliations:** 1 Institute for Physical Activity and Nutrition (IPAN), School of Exercise and Nutrition Sciences, Deakin University, Geelong, IC 3220, Australia; szeyen.tan@deakin.edu.au; 2 Department of Food Science and Human Nutrition, Michigan State University, 2110 S. Anthony Hall, 474 S. Shaw Ln, East Lansing, MI 48824, USA

**Keywords:** sweet taste, psychophysics, nutrition, diet, threshold, intensity, liking

## Abstract

Taste is frequently cited as an important factor in food choice, and while a number of studies have attempted to identify relationships between taste function and dietary intake, a systematic review of these studies has been lacking. This review identified studies that examined associations between taste function or taste perception and dietary intake. The purpose was to determine which taste measure was most closely associated with dietary intake in healthy adults. Studies that measured some component of dietary intake, either acutely or longer-term, were eligible for inclusion. Studies were grouped into three categories: those that measured sensitivity (thresholds), intensity, or hedonic responses to sweet stimuli. Sensitivity and intensity studies demonstrated little association with dietary intake measures. Hedonic measurements were more likely to be associated with dietary intake, especially if sweet likers were analyzed separately from sweet dislikers, but the degree of heterogeneity among stimulus concentrations and dietary measures as well as small sample sizes likely obscured more consistent relationships between hedonic evaluation and dietary intake. Due to the potential for within-day and between-day variability in both taste function and dietary intake, future work should explore obtaining more than one taste measurement before comparing results to longer-term dietary assessments and attempts to standardize methods.

## 1. Introduction

The sense of taste is commonly referred to as the “gatekeeper” of food intake [1]. This concept is supported by consumer surveys that report food choices are made primarily based on the flavor of the selected foods, with considerations about healthfulness or cost typically rated as less important [2]. Taste is an important component of the chemosensory attributes (taste, smell, chemesthesis or chemical irritation) that comprise flavor [3], and thus, guide food selection and intake. Dietary intake, in turn, influences nutritional status and body composition. Thus, individual differences in taste function and perception may lead to differences in dietary behaviors and risk of chronic disease [4].

Each taste quality has been associated with specific nutrients that are important to health and well-being. For example, sweet taste is commonly thought to help identify sources of carbohydrate, sour taste with the presence of vitamins, salty taste with essential electrolytes, and umami with protein [5]. Bitter taste likely serves as a warning against potentially dangerous compounds [5]. If these purported functions are accurate, then positive associations between taste function and/or preference for these taste qualities and related nutrient intake should exist. 

Research regarding taste is typically concerned with one of two questions. First, how well does the system function? Sensitivity testing, which involves determining the absolute minimum concentration of a stimulus that can be reliably detected (detection threshold) or recognized (recognition threshold), is frequently performed, but perceived intensity measurements of suprathreshold concentrations are also used. Threshold measurements can take several forms, but these tests usually involve presenting the participant with several samples – only one of which contains the stimulus of interest. The participant is required to identify the sample that contains the stimulus. A variety of approaches in terms of the number of samples to present and number of correct answers needed to stop the experiment exist [6]. Intensity measurements typically involve presenting a stimulus to the participant and asking for a rating of the intensity. Scales commonly used include a visual analog scale [7], a category scale [8], or a general Labeled Magnitude Scale [9]. The second question typically assesses a hedonic aspect, such as, how much is the stimulus liked, the preferred stimulus when a participant is asked to compare two or more stimuli of different concentrations, or the optimal stimulus concentration—often determined using an adjustment method where the participant increases or decreases the concentration of the taste quality. All of the taste measures just described are considered to be independent of each other, providing separate but complementary information about how the stimulus is detected and perceived [10]. 

When research is conducted on a specific taste quality, model stimuli, often consisting of a prototypical stimulus dissolved in deionized water, are typically used. For example, commonly used prototypical stimuli for sweet taste include sucrose or glucose solutions; whereas, sodium chloride solutions comprise the typical salty stimulus. Participants usually swish and then expectorate the liquid samples, but other approaches, including filter paper impregnated with stimuli [11], cotton swabs [12], edible wafers [13], or edible films [14] have been used. The simplicity of model systems allows for attention to be focused on the taste quality of interest with minimal distraction, but the obvious drawback of the model system is that it does not reflect the complex sensory experiences provided by foods and beverages. Thus, the question that arises is: how closely do taste test results using model systems correlate with dietary intake? 

Given their simplicity but seemingly limited ecological validity [15], the ability of taste tests using model solutions to adequately predict dietary intake was previously considered limited [16,17]. However, few studies had adequately assessed intake when this question was first considered [16]. The question remains relevant, as recent work has examined how results from taste testing are associated with dietary intake. For example, the proposal of “fat” as another taste quality has led to renewed interest in connecting taste measurements to dietary intake and weight status (for a recent meta-analysis, see [18]). This suggests that relationships between taste measures and intake remain of interest to taste researchers. 

In recent years, sugar intake has been proposed as a potential cause of the increasing prevalence of obesity globally [19,20]. The relationship is especially strong between intake of sugar-sweetened beverages and obesity [21]. As a result, recommendations that added sugar in habitual diets should not exceed 10% of total daily energy intake have been made by a number of governmental and non-governmental organizations including the United States Dietary Guidelines for Americans [22], the Australian Dietary Guidelines [23], and the World Health Organization [24]. Mechanistically, scientists posit that sugar consumption is driven by hedonics, i.e., its pleasant sweet taste, and evidence also suggests that sweet taste enhances the liking and wanting of sweet-tasting foods [25]. Some studies further demonstrated that sugar activates the opioid (e.g., nucleus accumbens) and dopaminergic (e.g., ventral tegmental area and right amygdala) reward centers in the brain [26,27], leading to the notion that sugar is ‘addictive’ and leads to excessive food intake and subsequent weight gain. Together, these mechanistic studies appear to suggest that sweet taste triggers food seeking behaviors and dietary intake. Although a number of individual studies have performed sweet taste testing using model systems and assessed associations with intake, to our knowledge, a systematic review summarizing these findings has not been undertaken. Therefore, the purpose of this review was to determine if psychophysical tests for sweet taste were associated with dietary intake and, if possible, to determine which test is the most closely associated with dietary intake. 

## 2. Materials and Methods 

A systematic literature search of the electronic databases PubMed, PsycInfo, Web of Science, and CINAHL was conducted. The search string used in PubMed was (“Taste” (Mesh)) AND (“Diet, Food, and Nutrition” (Mesh)); filters included Adult 19+, English, and Human. These filters were used in the other databases when available. Review articles that were identified were searched to identify articles that the searchers missed. Studies that recruited generally healthy individuals and collected at least one psychophysical measure of sweet taste and reported some sort of dietary intake measure, either acute or long-term were included. There was no restriction on adiposity, that is, all categories of body mass index were accepted. Studies were excluded if the populations were currently or had previously been ill, for example diabetes, alcoholism, or eating disorders; had known changes or deficits in chemosensory function, for example gastric bypass surgery patients; were pregnant; or were smokers. The review protocol was registered with PROSPERO, review #CRD42018111833.

After the initial searches were completed and duplicate entries removed, all potential studies were entered into a master database. Initial screenings by title and abstract were completed by the authors. In the case that a determination to include or exclude could not be made based on the abstract, the full paper was reviewed. The authors discussed questions about inclusion or exclusion until consensus was reached. The authors searched the reference lists of relevant articles to identify potential articles (*n* = 2) that were missed by the systematic search. 

## 3. Results

**Figure 1:**
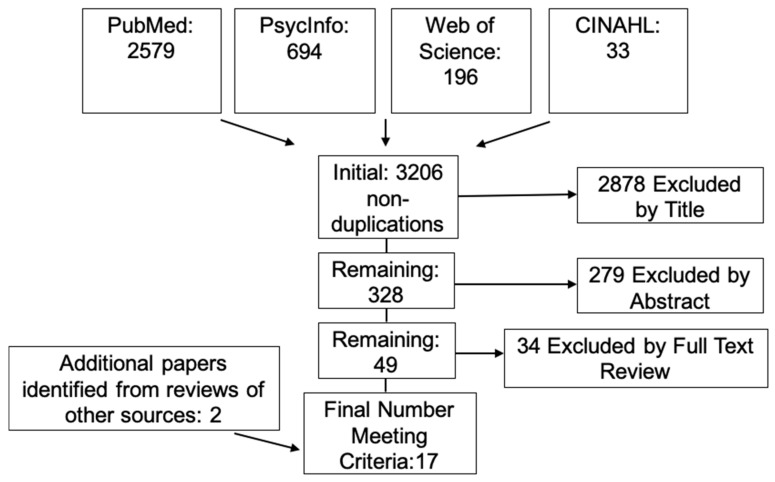
A total of 17 articles meeting the inclusion criteria were identified.

In total, 3206 publications were identified and 17 were included in this review (Figure 1). Studies were placed into three categories based on psychophysical method utilized: (1) sensitivity measurements consisting of detection and recognition thresholds (*n* = 6), (2) intensity measures (*n* = 8), and (3) hedonic evaluations, namely liking and preference (*n* = 13). Some studies used more than one method; those that did were examined multiple times. Given the heterogeneity of psychophysical measures [10] and stimuli concentrations [28] as well as differences in stimuli tested (glucose vs. sucrose vs. non-nutritive sweeteners) [29] and dietary intake assessment methods [30], a meta-analysis could not be attempted. 

### 3.1. Sensitivity Testing

A total of six studies examined relationships between taste sensitivity and dietary intake [9,16,29,31,32,33] (Table 1). Studies varied in terms of the stimuli used, e.g., glucose vs. sucrose vs. non-nutritive sweeteners, the ranges of concentration tested, and the dietary assessment methods employed. Sensitivity was measured based on detection threshold [9,29,31,32], recognition threshold [9,16,29], and/or ability to correctly identify a 9 mM sucrose solution three times in a row using a triangle test [33]; individuals who could perform this task correctly were classified as “highly sensitive”. Of the six studies identified, only two observed significant associations between sweet taste thresholds and dietary intake [32,33]. One of the studies (*n* = 30) was an acute experimental study that reported that individuals who were highly sensitive to a 9 mM sucrose solution consumed significantly less carbohydrate and more non-sweet foods, dietary protein, and protein as a percent of energy at an *ad libitum* feeding opportunity 60 min after exposure to either a sweet, non-sweet (umami), or “no-taste” soup [33]. The use of a 9 mM sucrose solution to establish sweet taste sensitivity is not an approach that was used by any other study in this review, and the validity of this approach has not been established. The second study (*n* = 56) reported that aspartame threshold was negatively associated with energy intake as assessed by a 7-day food diary [32]. However, the association was very weak, albeit statistically significant, and may have limited implications (beta coefficient = −0.003, *p* < 0.0009); no further association between sucrose threshold and any diet measures were observed. Another study examining non-nutritive sweetener thresholds did not identify diet-taste relationships [29]. Differences in diet assessment methods (FFQ [29] vs. 7-day food diaries [32]) could contribute to these disparate results.

To summarize, most available studies failed to observe a significant relationship between sweet sensitivity and dietary intake, suggesting that testing for sweet taste threshold is not likely to be predictive of dietary intake. The only studies that reported an association found that sweet-sensitive individuals consumed less carbohydrate and more non-sweet foods [33]. The methodological limitations and small samples sizes of these studies also limit the generalizability of the findings.

### 3.2. Intensity Testing

Eight studies examined relationships between measures of sweet taste intensity and dietary intake [7,9,16,29,34,35,36,37] (Table 2). As with the sensitivity studies, stimuli and concentrations tested also varied widely. Only two of the ten studies observed significant relationships [9,29]. The first study (*n* = 42) reported negative associations between diet and intensity ratings for a 250 mM glucose stimulus [9]. Intensity was negatively correlated with total energy, carbohydrate (starch as well as total sugar, glucose, and fructose), but not sucrose intake. Sweet food intake was also negatively associated with intensity ratings of the 500 mM and 1000 mM samples. In this study, dietary intake was measured both by 4-day weighed food records as well as by an unvalidated sweet food FFQ and a sweet beverage liking questionnaire. The second study (*n* = 60) reported that intensity ratings for Rebaudioside A and sucralose, both non-nutritive sweeteners, were positively associated with mean total energy intake (*p* < 0.01 for both) [29]. No associations between intensity ratings and other dietary measures, including carbohydrate, sugar, or starch were observed, and no associations with the other sweet stimuli tested (glucose monohydrate, fructose, sucrose, or sucralose) were noted [29]. This study relied on the validated Cancer Council of Victoria Food Frequency Questionnaire [38] to assess dietary intake. 

In conclusion, only two studies demonstrated the utility of sweet taste intensity ratings in reflecting dietary intake, and neither study used sucrose—a prototypical sweet taste stimulus. The negative association between sweet taste intensity rating of glucose and energy as well as carbohydrate intake was consistent with the findings from the sensitivity studies that also reported significant negative associations [9,29]. On the other hand, associations with non-nutritive sweeteners (Rebaudioside A and sucralose) were present but positively associated with dietary intake. Further study is needed to understand the underlying mechanisms that contribute to these distinct relationships. 

### 3.3. Hedonic Testing

A total of 13 papers examined relationships between hedonic evaluation and dietary intake [7,8,9,16,28,31,34,36,37,39,40,41,42]. As before, the concentrations of sweet solution used in these studies varied considerably as did dietary assessment methods (Table 3). In contrast to the sensitivity and intensity studies, all but one [9] used sucrose. Hedonic measurements included determining the preferred concentration out of a range of stimuli [31] or through an adjustment task [16,42] or a rating of how much the stimulus was liked, typically using either a visual analog [7,28,37,40,41], labelled magnitude scale [9,34,36], or likert-style hedonic scales [8,39]. Five of the studies that measured hedonics also classified participants as sweet “likers” or “dislikers” [28,34,37,40,41]. A sweet liking phenotype has been associated with different hedonic responses to sweetness (for a recent review, see [37]), so failure to identify sweet liker phenotype could influence findings. That is, if the study population was comprised predominantly of sweet likers or dislikers, results could be skewed. Therefore, these studies are presented separately from the others. One study analyzed the data with and without sweet liker classification [37], so it is reported twice – both with those studies that did and did not identify sweet likers. 

#### 3.3.1. Studies that Determined Sweet Liking Phenotypes

Among the five studies that distinguished between sweet likers and dislikers, the classification methods used to determine sweet liker status varied greatly [28,34,37,40,41]. Classification was performed by hierarchical cluster analysis [28,41]; by preferred concentration cut-off, i.e., favorable ratings above a specific concentration [34,40]; a mean favorable rating over all concentrations tested [41]; and a pattern of increasing hedonic scores [37]. Among these six papers, three observed relationships between hedonics and dietary intake measures [28,37,40]. Among the studies demonstrating associations with sweet liker status and intake, one (*n* = 418) reported that energy intake from sugar-sweetened beverages was higher among likers compared to dislikers (*p* = 0.008) based on a beverage food frequency questionnaire [28]. A second study (*n* = 196) that examined sweet liker and PROP taster status combinations observed that individuals who were both sweet likers and PROP tasters reported consuming more energy from beverages and fiber as measured by two 24-h recalls [40]. The last study (*n* = 132) reported positive associations between the preferred level of sucrose and frequency of sweet food consumption, intake of refined sugars, and total sugars [37]. Two studies did not observe taste-diet relationships, but the reported sample sizes raise questions about the power of these studies to detect relationships (*n* = 12 (6 sweet likers) [34] and *n* = 36 (12 sweet likers)) [41]. Overall, sweet likers appear to consume more energy from sugar-sweetened beverages and more energy from refined and total sugars. It appears that identifying an individual’s sweet liking phenotype may increase the likelihood that relationships between hedonic scores and dietary intake will be observed, especially if sample sizes are sufficiently large enough. 

#### 3.3.2. Studies that Did Not Determine Sweet Liking Phenotypes

Among the nine studies that did not classify sweet likers, associations between hedonic responses and intake were observed in five [9,16,31,37,42] but not in the other four [7,8,36,39] (Table 3). Preferred sweetness concentration was associated with greater total energy intake [31], carbohydrate intake [31,42], percent of sweet calories consumed [37,42], refined and total sugars [37], and frequency of carbohydrate-rich food selections [42], while one study observed positive associations with liking ratings of glucose at 500 mM and 1000 mM and total energy and carbohydrate (total sugar, fructose, glucose) but not starch and sucrose intake [9]. One study observed a negative association between preferred sweetness concentration and carbohydrate intake [16]. The studies finding associations between hedonic evaluations and dietary intake used one 24-h recall [31], 4-day weighed food records [9], and 7-day diet records [16,42]. Sample sizes for these studies ranged from *n* = 25 [42] to *n* = 51 [31]. Studies not observing associations reported sample sizes ranging from *n* = 17 [8] to *n* = 100 [7]. In summary, hedonic measures appear to be better correlated with dietary intake, and these relationships are strengthened when sweet likers are analyzed separately. 

## 4. Discussion

The sensory properties of food, including taste, play an important role in food selection and intake [2]. Psychophysical studies exploring taste function and perception have sought to determine if responses obtained in these studies can be associated with dietary intake. Given the challenges of assessing dietary intake [43], a proxy measure that is a simple, quick, and reliable predictor of intake would be welcomed.

Of the taste testing methods used—sensitivity testing, intensity measures, or hedonic evaluation—hedonic ratings proved to be superior in their ability to correlate with dietary intake, although these studies also did not report consistent findings. The fact that sensitivity was not a reliable indicator of dietary intake was not unexpected, as others have noted that an individual’s sensitivity to a taste quality often fails to predict intake since these exposures can be quite dissimilar to the suprathreshold exposures experienced while eating [16,44]. Intensity measures lacked predictive power as well. One study observed positive associations between dietary intake and hedonic evaluation but not with intensity [37]. Another study reported that intensity evaluations between sweet likers and dislikers did not differ [28]. These results further support the argument that measuring sensitivity, intensity, and hedonic responses provides distinct but complementary information about the taste sensations experienced by an individual [10], but that, based on the available data, hedonic evaluation may provide a more reliable indication of dietary intake. 

Further, among the studies that classified sweet likers and dislikers, three of the five studies reported that sweet likers were more likely to demonstrate associations between dietary intake measures and hedonic evaluations. Sweet likers are typically classified by increasingly favorable hedonic responses to increasingly sweeter stimuli [45]. Thus, the positive associations between hedonic responses and intake of sugar sweetened beverages and sugar intake make intuitive sense. The two studies [34,41] that failed to see associations between hedonic responses and intake in sweet likers had small sample sizes of sweet likers (*n* ≤ 12). Intriguingly, while the methods used to assess sweet liking phenotype differed, results were consistent across studies. This agrees with others who reported that among these methods, no single classification approach demonstrated superiority [45]. 

The differences in both taste and diet measurements likely contribute to the discrepancies reported. First, a discussion of the taste measurement differences. The stimuli and concentrations used will have a direct impact on results. While different nutritive sweeteners were noted to have detection and recognition thresholds as well as intensity scores that were correlated with each other, actual values differed [46]. This is unsurprising, as different sugars have different potencies; sucrose, for example, is sweeter than glucose at the same concentration [47]. Further, the human sweet receptor responds to many compounds besides mono- and disaccharides, including amino acids, proteins, and non-nutritive sweeteners [48]. Sucrose and glucose are presumed to be the best stimuli to correlate with dietary intake, but this has not been tested, and one study reported that the threshold for the non-nutritive sweetener aspartame was negatively associated with energy intake, unlike sucrose [32]. The concentrations of the sweet stimulus presented to a participant can also influence taste results. Smaller differences between successive concentrations will allow for more precise determination of the taste threshold, but additional trials add to participant burden and increase the risk of fatigue. There is no standardized procedure for determining the difference in concentration between one stimuli and the next. The range of concentrations presented to participants in order to determine sweet liker/disliker phenotypes also varied by study [28]. It is conceivable that some individuals could be classified as sweet likers with one set of concentrations and sweet dislikers if the concentrations presented were higher. This is especially true if sweet liker phenotype is determined by the response to one concentration. Thus, if individuals were misclassified, results could change. 

In terms of dietary assessment, it is well known that self-reported dietary information is subject to over- and under-reporting [49]. Over- or under-reporting could obscure taste-diet relationships. In addition, due to the high degree of variability in intake from one day to the next, depending on the nutrient of interest, many days of intake in the form of diet diaries or records must be recorded [50]. For example, at minimum, two weeks of intake records are needed to estimate average energy intake in an individual, which is impractical for many studies, and accuracy declines over time [51]. This number falls to three days when estimating energy intake for groups of people [50]. Even with this reduction, dietary record keeping can be burdensome for participants [43] and items consumed can be poorly estimated or forgotten entirely. 

There are two main approaches to reduce participant burden when assessing dietary intake. These include the 24-hour diet recall, where participants are asked to remember what they ate during the previous day rather than recording it as each food and beverage is consumed, or a food frequency questionnaire (FFQ) [43]. The 24-h recall allows dietary information to be recorded at one time point, but accurate information collection relies on trained staff and suffers from recall bias [43]. FFQs employ a checklist approach, where participants can indicate how much and/or how often they consume certain foods. The main drawback of this approach is that the ability to accurately remember and quantify intake is severely compromised [43]. While both approaches are valuable, diet diaries are considered to be more accurate measures [43]. 

Based on the studies examined, there was no clearly superior method of dietary assessment that was more likely to identify taste-diet relationships. For the sensitivity studies, among the studies observing relationships, one utilized an acute intake measurement, i.e., consumption following a pre-load [33], while the other used 7-day food diaries [32]. Studies not observing relationships between taste sensitivity and dietary intake relied on 4-day weighed food records [9], food frequency questionnaires [9,29], 24-hour recall [31], and 7-day food diaries with predominant taste recorded [16]. For intensity, studies that observed relationships between taste and diet used 4-day weighed food records as well as an unvalidated sweet food FFQ and a sweet beverage liking questionnaire [9] and a validated FFQ not used by any other of the studies included in this review [29]. Studies failing to find associations between intensity measures and diet used two 24-h food recalls [7], multiple (3–14) day diet records [16,34,35,39], *ad libitum* intake of specific test foods [8], and various food frequency questionnaires [35,36,39]. Studies measuring hedonic responses that observed associations used multiple day (3–7) food records [9,16,42], 24-hour recalls [31,40], and food frequency questionnaires [9,28]. Studies that did not find associations used multiple day (3–14) food records [34,39], food frequency questionnaires [36,41], 24-h recalls [7], and food preference surveys [39]. At this time, it is not possible to make a recommendation for one dietary assessment method over the other. 

The majority of the studies relied on a one-time measure of taste response and attempted to map this response to dietary intake that spanned over days or months—a further limitation of the literature. Taste responses can vary throughout the day [52] or across days [31], posing problems in terms of test-retest reliability [53]. Day-to-day variability in both taste responses and dietary intake could obscure more immediate or acute relationships. One study noted that taste-diet relationships were observed after a night of sleep that lasted less than 7 h but saw no relationships after a night of longer sleep [31]. Sleep or other confounding variables may obscure taste-diet relationships. One of the two studies that did assess acute intake observed that sweet taste sensitivity correlated with a greater amount of non-sweet foods, protein, and protein as a percent of energy consumed by highly sensitive participants, and those participants also consumed less carbohydrate as a percent of energy [33]. The other study that assessed acute intake observed no relationships between intensity and hedonics [8]. The selection of the foods available for *ad libitum* intake could influence intake; thus, in addition to the different taste measures, it is difficult to compare these studies. Further exploration of whether taste measures are superior predictors of acute intake compared to longer-term intake needs to be undertaken.

There are several limitations to this review. As with all systematic reviews and meta-analyses, the inclusion criteria dictate the findings. While all studies were considered, taste testing studies are at high risk of bias due to the reliance on non-random selection of subjects and failure or inability to blind researchers and participants to the test stimuli or purpose of the study. The decision to focus solely on sweet taste limits generalizability to other taste qualities. The heterogeneity of taste testing and dietary assessment methods makes definitive conclusions difficult. Further work examining taste-diet relationships in children and populations with chronic conditions should be undertaken.

## 5. Conclusions

In conclusion, only a small proportion of available studies reported significant associations between taste sensitivity, intensity, and hedonics with dietary intake. However, of those that reported significant associations, sensitivity and intensity measurements (sensory function) were negatively associated with intake, while liking and preferred concentration measurements (hedonics) were positively associated with intake in all but one study. Measures of taste liking and preference appear to provide relatively superior insight into dietary behaviors compared to sensitivity and intensity measures. Future considerations regarding standardizing methods are imperative.

## Figures and Tables

**Table 1:** Sensitivity Studies Examining Taste-Diet Relationships.

Author (Year)	Subjects	Taste Test	Sweet Stimuli	Stimuli Concentrations	Dietary Assessment Methods	Key Findings
Mattes (1985) [16]	*n* = 35 (17 M, 18 F) Age = 18–42 years old	RT	Sucrose	Serial half dilutions of sucrose: 1.2 × 10^−5^ M to 0.8 M	7-day diet record with predominant taste recorded	Sweet taste threshold and intensity did not correlate with sweet E, CHO, PRO and fat intake.
Martinez-Cordero (2015) [32]	*n* = 56 (30 M, 26 F) Age = 32.9 ± 7.9 years old	DT	Sucrose Aspartame	Sucrose—14 [ ] from 4.09 × 10^−1^ M to 1.63 × 10^2^ M Aspartame—14 [ ] from 0.82 × 10^−3^ M to 3.27 × 10^−1^ M Both at 0.2 log dilutions per successive solution	7-day food diaries	Aspartame threshold was negatively associated with E intake (B = −0.003 ± 0.001; *p* < 0.0009). No association between sucrose threshold and dietary intake.
Low (2016) [29]	*n* = 60 Age = 26.5 ± 1.0 years old	DT; RT	Glucose mono-hydrate Fructose Sucrose Sucralose Erithritol Rebaudio-side A	Varying concentrations for each	Validated FFQ; also assessed consumption of foods and/or beverages sweetened with high-intensity sweeteners	No association between threshold measures and dietary measures.
Smith (2016) [31]	*n* = 51 (9 M, 42 F) Age = 25 ± 8y	DT	Sucrose	2.1% *w*/*v* sucrose Quarter-log step dilutions	24-hour recall	No association between threshold measures and dietary intake.
Han (2017) [33]	*n* = 30 (16 M, 14 F) Age = 24–34 years old (M), 20–37 years old (F)	Sensi-tivity	Sucrose	9 mM	*Ad libitum* intake after soup preload (one sweet, one umami, one no-taste energy control)	Highly-sensitive consumed more non-sweet foods, PRO, %E from PRO, and %E from fat (after non-sweet soup only) (*p* < 0.05 for all). Highly-sensitive consumed less CHO as %E (*p* = 0.02).
Jayasinghe (2017) [9]	*n* = 42 (all F) Age = 28 ± 634 years old	DT; RT	Glucose	15, 30, 45, 60, 90, 120, 150, 180 mM	4-day weighed food record Sweet food FFQ Sweet beverage liking questionnaire	No association between threshold measures and dietary intake.

Abbreviations: [ ] concentration, CHO = carbohydrate, DT = detection threshold, E = energy, FFQ = food frequency questionnaire, F = female, M = male, PRO = protein, RT = recognition threshold, w/v = weight for volume.

**Table 2:** Intensity Studies Examining Taste-Diet Relationships.

Author (Year)	Subjects	Taste Test	Sweet Stimuli	Stimuli Concentrations	Dietary Assessment Methods	Key Findings
Mattes (1985) [16]	*n* = 35 (17 M, 18 F) Age = 18–42 years old	Intensity	Sucrose	5 concentrations ranging from 0.05 M to 0.80 M	7-day diet records	No association between intensity measures and dietary intake.
Holt (2000) [37]	*n* = 132, Australian 27 M, 42 F Malaysian 29 M, 34 F; Australian 22.8 ± 4.3 years old Malaysian 21.5 ± 1.2 years old	Intensity	Sucrose	2, 4, 8, 16 and 32% *v*/*v*	Separate FFQ for the Australian and Malaysian participants	No association between intensity measures and dietary intake.
Sartor (2011) [34]	*n* = 12 (7 M, 5 F) Age = 26 ± 6 years old	Intensity	Sucrose	0, −0.5, −0.75, −1, −1.25, −1.5, −1.75, −2, −2.25, −2.5, −2.75 log(sucrose) mol/L	14 diet diaries on random days	No association between intensity measures and dietary intake.
Cicerale (2012) [35]	*n* = 85 (89% F) Age = 21 ± 4 years old	Intensity	Sucrose	200 mM	Food & diet questionnaire Food variety survey 2 × 24-hour food diaries	No association between intensity and any diet measures.
Low (2016) [29]	*n* = 60 Age = 26.5 ± 1.0 years old (SEM)	Intensity	Glucose mono-hydrate Fructose Sucrose Sucralose Erithritol Rebaud-ioside A	Varying concentrations	Validated FFQ; also assessed consumption of foods and/or beverages sweetened with high-intensity sweeteners	Intensity and dietary intake associations varied by sweetener. Rebaudioside A and sucralose intensity ratings were positively associated with mean total E intake (*p* < 0.01 for both).
Stevenson (2016) [36]	*n* = 87 (38 M, 49 F) Age = 21 ± 3 years old (18–31 years old)	Intensity	Sucrose	0.03 M and 0.36 M	26-item Dietary Fat and Sugar questionnaire (DFS) designed to identify variation in saturated fat and added sugar intake	No association between intensity and any diet measures.
Jayasinghe (2017) [9]	*n* = 42 (all F) Age = 28 ± 6 years old	Intensity	Glucose	125, 250, 500, 1000 mM	4-day weighed food record Sweet food FFQ Sweet beverage liking questionnaire	Intensity at 250 mM or higher correlated negatively with total E, CHO (starch, total sugar, fructose, glucose) but not sucrose intake (*p* < 0.05 for all). Intensity also negatively associated with total sweet food intake (*p* < 0.05 for all).
Leong (2018) [7]	*n* = 100 (50 M, 50 F) Age = 25.7 ± 4.2 years old (M), 25.7 ± 5.1 years old (F)	Intensity	Sucrose	12.0% *w*/*v*	2 × 24-hour food recalls	No association between intensity and any diet measures.

Abbreviations: CHO = carbohydrate, E = energy, FFQ = food frequency questionnaire, F = female, M = male, v/v = volume for volume, w/v = weight for volume.

**Table 3:** Hedonic Studies Examining Taste-Diet Relationships.

Author (Year)	Subjects	Taste Test	Sweet Stimuli	Stimuli Concentrations	Dietary Assessment Methods	Key Findings
Weizenbaum (1980) [8]	*n* = 17 (5 M, 12 F) Age = 18.6 y (M), 19.7 years old (F)	Pleasantness	Sucrose	0.01, 0.023, 0.046, 0.1, 0.23, 0.46, 1.0 M	*Ad libitum* intake of salted peanuts and candies after testing	No relationship between pleasantness and amount of food consumed.
Mattes (1985) [16]	*n* = 35 (17 M, 18 F) Age = 18–42 years old	Preferred concentration of sweetness	Sucrose	Self-adjusted (dilution)	7-day diet records	Preferred concentration of sweet solution negatively correlated (*r* = −0.36, *p* = 0.04) with CHO intake.
Mattes (1986) [42]	*n* = 25 (all M) Age = 17–34 years old	Preferred concentration of sweetness using an adjustment task	Sucrose	0M & 1.0 M solutions were provided. Subjects modified the samples until the preferred sweetness was reached. Preferred sweetness levels from both the unsweetened and sweetened baseline stimuli were averaged.	7-day diet records	Mean preferred concentration was positively correlated with %CHO intake (*r* = 0.637, *p* < 0.001). Preferred concentration of the 1.0 M sucrose samples were positively correlated with %CHO intake (*r* = 0.748, *p* < 0.001), % sweet calorie intake (*r* = 0.504, *p* < 0.001), and frequency of selection of carbohydrate-rich foods (*r* = 0.532, *p* < 0.01).
Drewnowski (1999) [39]	*n* = 159 (all F) Age = 27.0 ± 0.7 years old (SEM)	Liking	Sucrose	5 [ ] ranging from 2% to 32% *w*/*v*	3-day food records; 171-item food preference checklist	No associations between liking and dietary intake measures, but higher hedonic ratings for sucrose were associated with higher ratings for sugar in tea and many sweet desserts.
Holt (2000) [37]	*n* = 132, separated into Australian-born Caucasian and Malaysian born; Australian: 27 M, 42 F Malaysian: 29 M, 34 F; Australian: 22.8 ± 4.3 years old Malaysian: 21.5 ± 1.2 years old	Liking	Sucrose	2, 4, 8, 16 and 32% *v*/*v*	Separate FFQs for the Australian and Malaysian subjects	Refined sugar intake was higher in sweet likers com-pared to dislikers. No other differences were observed. For all participants, positive associations between the preferred level of sucrose and frequency of sweet food consumption, intake of refined sugars, and total sugars were observed (*p* < 0.05).
Sartor (2011) [34]	*n* = 12 (7 M, 5 F) Age = 26 ± 6 years old	Pleasantness Preference	Sucrose	Pleasantness 11 [ ]: 0, −0.5, −0.75, −1, −1.25, −1.5, −1.75, −2, −2.25, −2.5, −2.75 log(sucrose) M Preference: 10 random presentations of pairs of 0, −0.5, −0.75, −1 and −1.25 log(sucrose) M	14 diet diaries on random days	No associations between taste measures and dietary intake.
Turner-McGrievy (2013) [40]	*n* = 196 (85% F) Age = 42.6 ± 11.0 years old	Liking	Sucrose	0.05, 0.10, 0.21, 0.42, and 0.83 M. Participants who liked the 0.83 M sucrose solution the best were classified as sweet likers	2 × 24-hour food recalls	Those who were sweet likers consumed more E from beverages and less fiber (*p* < 0.05).
Methven (2016) [41]	*n* = 36 (12 M, 23 F, 1 unknown) Age = 26 years old (median)	Liking	Sucrose	3%, 6%, 12%, 24%, 36%	FFQ used by EPIC	Intake did not differ between sweet likers and dislikers.
Smith (2016) [31]	*n* = 51 (9 M, 42 F) Age = 25 ± 8 years old	Preferred concentration of sweetness	Sucrose	2.1% *w*/*v* stock solution Quarter-log step dilutions	24-hour recall	Sweet preference after short-sleep was positively correlated with E intake (*r* = 0.31, *p* = 0.043) and CHO intake (*r* = 0.32, *p* = 0.34), but not after habitual sleep.
Stevenson (2016) [36]	*n* = 87 (38 M, 49 F) Age = 21 ± 3 years old (18–31 years old)	Liking	Sucrose	0.03 & 0.36 M	26-item Dietary Fat and Sugar questionnaire (DFS) designed to identify variation in saturated fat and added sugar intake.	No association between liking and any diet measures.
Jayasinghe (2017) [9]	*n* = 42 (all F) Age = 28 ± 6 years old	Liking	Glucose	125, 250, 500, 1000 mM	4-day weighed food record; Sweet food FFQ; Sweet beverage liking questionnaire	Sweet taste liking at 500 mM or higher correlated positively with total E, CHO (total sugar, fructose, glucose) (*p* < 0.05 for all) but not starch and sucrose intake.
Garneau (2018) [28]	*n* = 418	Liking	Sucrose	5 [ ] ranging from 0% *w*/*v* to 13.7% *w*/*v*	Validated beverage FFQ (BEVQ-15)	Mean E intake from all beverages was higher among likers compared to neutrals (*p* = 0.004). Total E intake by dislikers did not differ from the other groups. E intake from sugar-sweetened beverages was higher among likers compared to dislikers (*p* = 0.008). Neutrals did not differ from the other groups.
Leong (2018) [7]	*n* = 100 (50 M, 50 F) Age = 25.7 ± 4.2 years old (M), 25.7 ± 5.1 years old (F)	Liking	Sucrose	12.0% *w*/*v*	2 × 24-hour food recalls	No association with liking and dietary intake.

Abbreviations: [ ] = concentration, CHO = carbohydrate, EPIC = European Prospective Investigation into Cancer and Nutrition study, E = energy, FFQ = food frequency questionnaire, F = female, M = male, w/v = weight for volume.
